# Pulse Wave Velocity for Risk Stratification of Patients with Aortic Aneurysm

**DOI:** 10.3390/jcm11144026

**Published:** 2022-07-12

**Authors:** Wilma Schierling, Julia Matzner, Hanna Apfelbeck, Dirk Grothues, Renate Oberhoffer-Fritz, Karin Pfister

**Affiliations:** 1 Department of Vascular Surgery, University Hospital Regensburg, Franz-Josef-Strauß-Allee 11, D-93053 Regensburg, Germany; julia.matzner@klinikum-straubing.de (J.M.); hanna.apfelbeck@ukr.de (H.A.); karin.pfister@ukr.de (K.P.); 2 University Children’s Hospital of Regensburg (KUNO-Clinics), Franz-Josef-Strauß-Allee 11, D-93053 Regensburg, Germany; dirk.grothues@ukr.de; 3 Institute of Preventive Pediatrics, Technical University Munich, Georg-Brauchle-Ring 60–62, D-80992 Munich, Germany; renate.oberhoffer@tum.de

**Keywords:** abdominal aortic aneurysm (AAA), arterial stiffness, atherosclerosis, endovascular aneurysm repair (EVAR), Mobil-O-Graph, pulse wave velocity (PWV)

## Abstract

Background: Patients with an aortic aneurysm are at high cardiovascular risk. Pulse wave velocity (PWV) is used as a parameter for risk stratification but may be affected by aortic disease (AoD). This study aimed to investigate the dependence of PWV on treated or untreated AoD and to identify modifiable factors of PWV. Methods: The measurement of PWV with the Mobil-O-Graph was performed fully automatically in a collective of 381 patients (75.6% male and 24.4% female). Of all patients, 53.8% had nonaortic atherosclerotic vascular disease (AVD), 28.9% had treated AoD, and 17.3% had untreated AoD. Results: There was a statistically significant effect of age (R^2^ = 0.838) and current systolic blood pressure (SBP) on PWV (p_age corrected_ < 0.05). After correction for age, no statistically significant difference was found between the PWV of men and women, patients with different body weights or degrees of chronic kidney disease, diabetics and nondiabetics, and smokers and nonsmokers. Comparison between patients with nonaortic AVD and treated or untreated AoD revealed no statistically significant differences (PWV_nonaortic AVD_ 10.0 ± 1.8 m/s, PWV_treated AoD_ 10.0 ± 1.5 m/s, PWV_untreated AoD_ 9.8 ± 1.6 m/s; p_age corrected_ > 0.05). Conclusions: PWV determined with the Mobil-O-Graph correlated with age and current SBP. Neither aortic disease versus nonaortic AVD, its treatment, nor other cardiovascular risk factors had a significant effect on PWV. Successful blood pressure control is crucial to avoid high PWV and thus an increase in cardiovascular events.

## 1. Introduction

Arterial stiffness is a major contributor to cardiovascular disease leading to myocardial infarction, stroke, end-stage renal disease, and critical leg ischemia, and is a major cause of cardiovascular complications such as ventricular hypertrophy or failure and aneurysm formation or rupture [1,2,3]. The first consensus conference on arterial stiffness was held in Paris in 2000 [1]. Today, the determination of PWV is a Class IIa (Level of Evidence A) recommendation for measuring arterial stiffness and predicting future cardiovascular events beyond standard cardiovascular risk factors [3] (pp. 698, 702). The 2007 ESH/ESC guidelines on hypertension proposed a fixed PWV threshold of 12 m/s for increased cardiovascular risk [4] (p. 704). This value was changed to the current threshold of 10 m/s by expert consensus in 2012 [3] (p. 704). Measurements of PWV can be performed either invasively [3] or noninvasively by determining pulse transit time along an arterial segment [3,4] or by ultrasound and magnetic resonance imaging (MRI) techniques [5]. Carotid to femoral PWV (cfPWV) has become the gold standard (Class I recommendation; Level of Evidence A) [3] (p. 700) for routine PWV assessment and large-scale clinical trials [4]. Discrepancies between devices that measure the pulse wave at two points and calculate PWV between these points have been noted, particularly in distance estimation [5]. Validated oscillometric cuff devices such as the Mobil-O-Graph estimate PWV using a specific algorithm [6]. The advantages of the Mobil-O-Graph are its easy handling, even in an outpatient setting, and its ability to record PWV in addition to blood pressure over 24 h [6,7,8,9]. Studies using the Mobil-O-Graph on hemodialysis patients have shown that the 48 h PWV was the only vascular parameter independently associated with increased risk of cardiovascular events and mortality [10] and that disability was independently associated with higher PWV [11].

The mechanical properties of the aortic wall may be affected by aortic disease, resulting in decreased aortic distensibility and increased aortic stiffness in aneurysmal segments [12,13]. Studies have described significantly higher PWV in patients with aortic aneurysms compared with control subjects [12,14]. Changes have also been observed in patients after open and endovascular aortic aneurysm repair (EVAR). PWV increased after EVAR [15,16,17,18] and was significantly lower after open repair compared with EVAR [16,19].

A 2022 meta-analysis included a total of 13 out of 367 papers, 10 papers with patients after abdominal EVAR and 3 papers with patients after thoracic endovascular aortic repair (TEVAR). In four of these papers, open surgery was accepted as the EVAR comparator. Changes after open surgery were not found, but pooled data analysis was not possible. EVAR and TEVAR appeared to result in a significant increase in PWV and thus aortic stiffness, which would be important because of an increased risk for cardiovascular events. However, the data were clearly limited by heterogeneity and the small number of available studies, so robust evidence is currently not available [20].

Patients with aortic aneurysms are at high cardiovascular risk [21,22]. Several studies have compared PWV in patients after endovascular aortic repair with matched but healthy controls [17,18]. Measurement of aortic PWV might be influenced by aortic disease itself and thus lose validity. The aim of the present study was to compare the patients with aortic disease with the group of nonaortic AVD patients to further clarify the dependence of noninvasively measured PWV on AoD and to determine modifiable factors of PWV. Here, the measurements were performed with the fully automatic oscillometric cuff device Mobil-O-Graph (IEM, Stolberg, Germany).

## 2. Materials and Methods

### 2.1. Study Design and Recruitment

The present study is a single-center registry analysis of a prospectively kept database at the University Hospital Regensburg. Between September 2015 and March 2016, 500 consecutive patients with AoD and nonaortic AVD presenting to our hospital as outpatients or inpatients were included.

Data were collected in accordance with the Declaration of Helsinki. The study was approved by the local ethics committee with the number 14-17-101. Participants gave informed consent for data acquisition and analysis.

**Figure 1:**
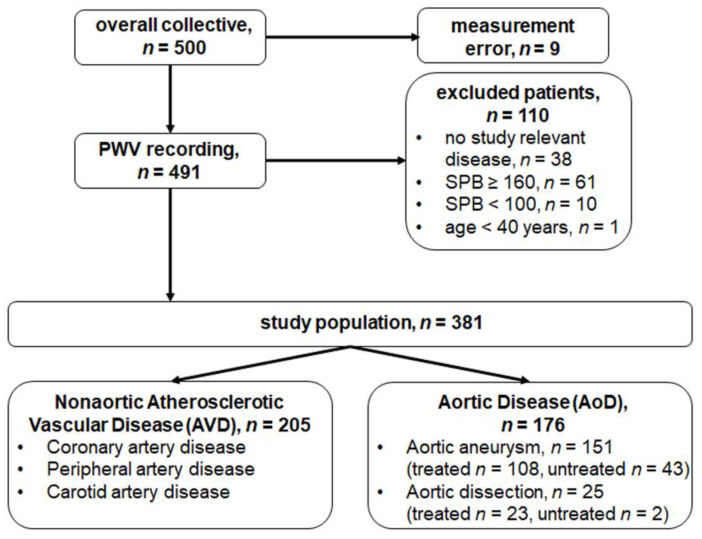
Flowchart for selection of study population.

PWV measurement was performed during routine clinical practice. All patients with nonaortic AVD such as coronary artery disease, peripheral artery disease, and carotid artery disease and all patients with aortic disease such as an aortic aneurysm or aortic dissection with systolic blood pressure ≥ 100 mmHg and <160 mmHg and age ≥ 40 years were included. Patients with venous vascular diseases such as thrombosis, varicose or post-thrombotic syndrome, blood pressure outside of the study range, and/or age < 40 years were excluded. Figure 1 shows the flowchart for study population selection. Type of vascular disease and treatment were recorded, as were age, gender, height, weight, smoking, medications, and diabetes. The eGFR (estimated glomerular filtration rate) was determined in the laboratory, and the current systolic blood pressure was measured with the Mobil-O-Graph.

### 2.2. Pulse Wave Velocity Recording

PWV was determined with the Mobil-O-Graph (IEM, Stolberg, Germany) on both upper arms of the patients in a supine position after five minutes of rest as a double measurement. The Mobil-O-Graph is an easy-to-use, fully automatic oscillometric device that is applied to the upper arm like a blood pressure cuff. All measurements were performed by a single, specially trained person. The patients were instructed not to move or speak during measurement. The measurement with the best data quality of the left arm was used for further analysis. If there was evidence of a relevant left subclavian artery stenosis due to a decreased SBP, the measurement with the best data quality of the right arm was used. In patients with an arteriovenous fistula, axillary lymph node dissection, or ongoing intravenous drug administration, the measurement was performed on one arm only. No PWV measurements were taken on the day of surgery itself or the first postoperative day.

### 2.3. Statistical Analysis

Statistical analysis was carried out using SPSS software (SPSS Inc., Chicago, IL, USA, version 24.0). Correlations were calculated using Pearson’s correlation coefficient r. Fisher’s transformation r-to-z was performed to compare correlation coefficients. Regression analysis was applied to determine the strength and direction of a relationship between two quantitative variables on the dependent variable PWV. A single or multi-factor analysis of covariance (ANCOVA or MANCOVA) was used to calculate the level of significance when examining associations or differences between the dependent variable PWV and at least one independent influencing variable. Here, the dependent variable *age* was controlled as a confounding variable in order to obtain as accurate a value as possible for the influence of the independent variable on PWV. In the case of a positive significant value of the independent variable, a post hoc test was conducted to determine the direction of significance. This difference was presented in the 95%-confidence interval (CI_95%_). The significance level was set at *p* < 0.05. Values are given as mean ± standard deviation (SD).

## 3. Results

### 3.1. Study Group

A total of 381 patients (75.6% male and 24.4% female) were included. Nonaortic AVD was present in 205/381 patients (53.8%) and AoD in 176/381 patients (46.2%), including 151/381 patients (39.6%) with aortic aneurysm and 25/381 patients (6.6%) with aortic dissection. In 108/151 patients (71.5%) with aortic aneurysm, the aneurysm had already been surgically treated. Of these patients, 82/108 (75.9%) had abdominal aortic aneurysms, and EVAR was the preferred treatment method in 93.5% of the cases. The median time since aneurysm exclusion was 2 years (<0.5 years: 30.6%, 0.5–4.9 years: 39.8%, 5.0–9.9 years: 22.2%, >10.0 years: 7.4%). At study inclusion, 43/151 (28.5%) aneurysm patients were surgically untreated and undergoing the best medical treatment therapy. Table 1 shows the demographic characteristics and cardiovascular comorbidities of all patients.

### 3.2. Age, Gender, and Recorded PWV

**Figure 2:**
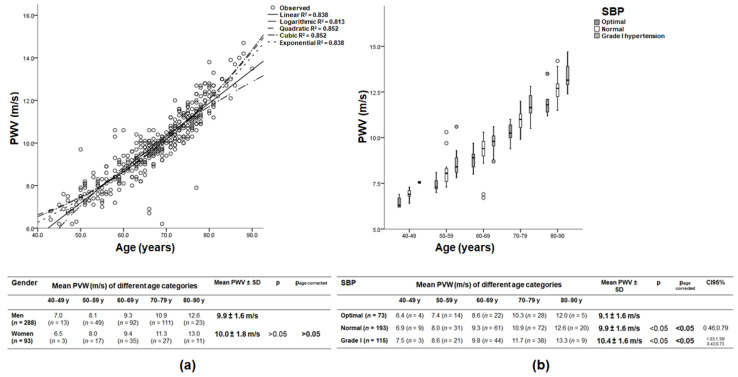
(**a**) PWV of study population showing the increase in PWV with age. Regression lines denote the results of the different regression analyses for age. The correlation coefficient R^2^ is given for each function. The quadratic and cubic functions are slightly more accurate than the linear function but can all be used; *n* = 381. There was no statistically significant difference in PWV between men and women (*p* > 0.05). y: years. (**b**) Boxplots of PWV as a function of age for different blood pressure categories. There was a statistically significant effect of current systolic blood pressure on PWV (*p* < 0.05). Horizontal lines indicate medians and the circle indicates outliers; SPB: systolic blood pressure; y: years; *n* = 381.

A comparison of the PWV values of both arms showed no statistically significant difference (PWV_right arm_ 9.9 ± 1.7 m/s and PWV_left arm_ 9.9 ± 1.7 m/s; *p* > 0.05). The PWV of the left arm was used for further evaluation as long as there was no suspicion of upstream stenosis. There was a statistically significant correlation between age and PWV, demonstrating higher PWV values for older patients (R^2^ = 0.838, *p* < 0.05; CI_95%_ = [0.15; 0.16]; Figure 2a). Due to the influence of age on PWV, all further results were finally compared after correction for age. The mean age of all patients was 67.5 ± 9.7 years (range 43–90 years). Men were, on average, 67.5 ± 9.5 years old and women 67.7 ± 10.4 years. There was no statistically significant difference in PWV between men and women (PWV_men_ 9.9 ± 1.6 m/s and PWV_women_ 10.0 ± 1.8 m/s; *p* > 0.05 and p_age corrected_ > 0.05; Figure 2a).

### 3.3. Systolic Blood Pressure and Recorded PWV

Depending on the current systolic blood pressure, patients were divided into 3 groups for analysis, namely *optimal* with SBP 100–119 mmHg, *normal* with SBP 120–129 mmHg, and *grade I hypertension* with SBP 140–159 mmHg. There was a statistically significant effect of the determined systolic blood pressure on PWV (PWV_optimal SBP_ 9.1 ± 1.6, PWV_normal SBP_ 9.9 ± 1.6 and PWV_grade I hypertension_ 10.4 ± 1.6 m/s; *p* < 0.05 and p_age corrected_ < 0.05; Figure 2b).

### 3.4. Cardiovascular Risk Factors, Chronic Kidney Disease (CKD), and Recorded PWV

For further analysis, the study population was divided into *normal weight* (BMI < 25 kg/m^2^), *overweight* (BMI 25–29.9 kg/m^2^), and *obese* (BMI ≥ 30 kg/m^2^) patients. A further distinction was made between diabetics and nondiabetics, smokers and nonsmokers, and patients with CKD stage 1/no CKD (eGFR ≥ 90), CKD stage 2/3 CKD (eGFR 30–89), and CKD stage 4/hemodialysis (eGFR < 30). While there were no statistically significant differences in PWV for BMI and diabetes, there appeared to be significant differences in PWV for smoking and different degrees of chronic kidney disease (Table 2). However, this was due to the fact that smokers were, on average, 5 years younger than nonsmokers (63.6 ± 8.6 versus 68.7 ± 9.7 years), and patients with an eGFR ≥ 90 (62.9 ± 8.1 years) were, on average, 5–7 years younger than patients with an eGFR of 30–89 (69.6 ± 9.4 years) or an eGFR < 30 (67.3 ± 11.3 years). After correction for age, no statistically significant PWV differences were found between smokers and nonsmokers and patients with different degrees of CKD (p_age corrected_ > 0.05; Table 2).

### 3.5. PWV of Patients with Aortic Disease and Nonaortic Atherosclerotic Vascular Disease

**Figure 3:**
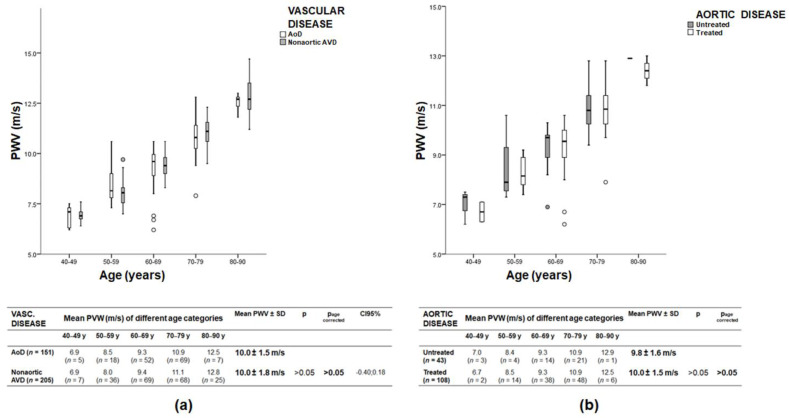
Boxplots of PWV as a function of age for patients with aortic disease (AoD) and nonaortic atherosclerotic vascular disease (AVD), *n* = 356 (**a**), and for patients treated or untreated for aortic disease, *n* = 151 (**b**). There were no significant differences between these groups. Horizontal lines indicate medians and the circle indicates outliers; y: years. Patients with aortic dissection were not included in this analysis due to the small number of cases.

The PWV of patients with aortic aneurysms (AoD, *n* = 151) was compared to the PWV of patients with coronary artery, peripheral artery, and/or carotid artery disease (nonaortic AVD, *n* = 205). Patients with aortic dissections were excluded from this comparative analysis due to the small number of cases (*n* = 25). The two groups of nonaortic AVD and AoD were comparable in age and systolic blood pressure (67.8 ± 10.0 years compared to 68.5 ± 8.5 years, and 132.9 ± 13.1 mmHg compared to 131.1 ± 14.5 mmHg, *p* > 0.05). The proportion of women was slightly higher in the group with nonaortic AVD than in the group with AoD (61/205, 29.8% compared to 24/151, 15.9%). There were no statistically significant differences between patients with nonaortic AVD and AoD (PWV_nonaortic AVD_ 10.0 ± 1.8 m/s and PWV_AoD_ 10.0 ± 1.5 m/s; *p* > 0.05 and p_age corrected_ > 0.05; Figure 3a). Comparison of patients with treated (108/151, 71.5%) and untreated (43/151, 28.5%) aortic aneurysm also showed no statistically significant difference in PWV (PWV_treated AoD_ 10.0 ± 1.5 m/s and PWV_untreated AoD_ 9.8 ± 1.6 m/s; *p* > 0.05 and p_age corrected_ > 0.05; Figure 3b).

## 4. Discussion

Arterial stiffness assessed by the PWV has been established as an independent risk factor for cardiovascular disease [2,3,4,23,24,25] and allows the identification of high-risk populations for more aggressive management of cardiovascular risk factors to prevent cardiovascular events [24]. The development of various methods for non-invasive measurement of PWV has enabled routine clinical evaluation of patients [4]. Methods for determining the time of travel of the pulse wave over a distance include devices that use applanation tonometry (e.g., SphygmoCor, PulsePen), piezoelectric mechanotransducers (e.g., Complior), cuff-based oscillometry (e.g., Arteriograph, Vicorder, Mobil-O-Graph), photodiode sensors (e.g., pOpmètre), and devices assessing brachial-ankle pulse wave velocity and cardiac-ankle PWV [2,3,5]. Oscillometric cuff devices, such as the Mobil-O-Graph, record brachial blood pressure and brachial waveforms to estimate central aortic pressure and PWV by a proprietary algorithm [3,26]. Calculation of PWV and central aortic pressure by this oscillometric device has been shown to have acceptable accuracy compared to invasive and non-invasive readings [6,27,28,29,30]. The current gold standard is the determination of the carotid to femoral PWV [4,26]. In a study by Salvi et al., 2019, seven non-invasive devices were compared with each other and with the invasive determination of aortic pulse wave velocity. The devices used to determine cfPWV showed strong agreement between each other and the invasive measurement [26]. Known problems of the non-invasive methods are, for example, that the measurement points—carotid and femoral—are not in the same arterial line and that an exact measurement of the arterial distance is not possible [1,3,5]. Thus, studies showed significant differences in PWV between different methods and/or patient groups with the recommendation to establish reference values for each of these techniques and groups (e.g., children, adolescents, adults, females, males) [2,4,5,25].

In recent years, the Mobil-O-Graph has proven to be a valid device for easy and also ambulatory determination of PWV [10,11]. In dialysis patients, for example, an independent association of PWV with an increased risk of cardiovascular events and mortality was found [10]. Comparison with the SpyhgmoCor showed acceptable agreement for aortic SBP and augmentation index (AIx75). PWV was slightly underestimated by the Mobil-O-Graph [30]. Patients with aortic aneurysms have an increased risk of coronary artery disease compared to the general population [21,22]. Effects of aneurysm geometry and stiffness on PWV have already been demonstrated by simulations of pulse wave propagation [31]. The presence of an aneurysmatic sac and variations in the sac modulus affect the propagation of the pulse waves qualitatively and quantitatively [31]. Several studies have addressed PWV in aneurysmal disease. The results have not always been consistent. Significantly higher PWV and lower distensibility were found in patients with a dilated thoracic aorta and abdominal aortic aneurysm [12,14]. Four weeks after EVAR, cfPWV increased significantly, whereas ba(brachialankle)PWV did not change significantly [15]. After open repair, PWV was significantly lower than after EVAR and decreased by 0.2 (±4.9) cm/s compared to preoperatively [16]. Others showed that the preoperative PWV did not correlate with the AAA diameter and that aortic PWV increased significantly six months after open aneurysm repair [13]. According to the most recent meta-analysis from 2022, aortic stiffness is significantly increased after endovascular aortic repair but not after open surgery. Due to heterogeneity and the small number of included studies, reliable evidence is not yet available to draw further conclusions from this analysis [20]. The influence of different blood pressure values on PWV, even in the same patient, is not discussed.

In other studies, matched but healthy subjects served as controls for patients after aortic stent-graft implantation [17,18]. For example, in the study by Tzilalis et al., 2012, the SBP of TEVAR patients was significantly higher than that of matched but healthy controls (134.10 versus 121.36 mmHg) [17]. In our study, patients with aortic disease and nonaortic atherosclerotic vascular disease were compared to further clarify the dependence of noninvasively measured PWV on aortic disease and determine modifiable factors of PWV. PWV was recorded oscillometrically using the Mobil-O-Graph. There was no statistically significant difference between patients with aortic disease and patients with nonaortic AVD. There was also no significant difference between patients with treated and untreated aortic aneurysms. EVAR was the preferred treatment method in 93.5% of the cases. The median time since aneurysm treatment was 2 years. The known, significant dependence of the PWV on age was also found in our study population. To avoid the influence of blood pressure on the results, only patients with systolic blood pressure between ≥100 mmHg and <160 mmHg were included. Nevertheless, even in this range, a significant influence of the currently determined SBP on PWV was demonstrated. Thus, blood pressure control is essential to avoid high PWV values. Other factors, such as gender, body weight, diabetes, smoking, or kidney disease, showed no significant effect on PWV after correction for age. If the data had not been age-corrected, misinterpretations would be possible. Approximately half of all patients with vascular disease in this study (188/381, 49.3%) had a PWV below the 10 m/s threshold, supporting the suggestion of age-dependent percentiles rather than a fixed PWV threshold.

A review by O’Rourke et al., 2002, on definitions of reference values already stated that all arterial stiffness values are dependent on blood pressure and that all reference values must be given as a function of age [1]. Otherwise, there is a risk of misinterpretation of the results. The requirement to provide reference values depending on age and blood pressure is at least discussed [4,25]. The AHA guidelines recommend recording mean arterial pressure and heart rate as possible confounders of PWV data (Class I recommendation; Level of Evidence B) [3] (p. 710). A large study published in the European Heart Journal 2010, which established reference values for 11,092 individuals without overt CVD, diabetes, or antihypertensive or lipid-lowering medication, showed a significant increase in carotid-femoral PWV with age and blood pressure categories [4]. The value of a fixed PWV threshold was therefore questioned. Instead, a comparison with specific age- and blood pressure-dependent percentiles of a reference or normal population was suggested for individual risk stratification [4]. Some data from recent years have questioned the suitability of the Mobil-O-Graph for PWV measurement [23,26]. Schwartz et al., 2019, examined the association between PWV recorded with the Mobil-O-Graph and age and systolic blood pressure and compared the results with the cfPWV. Age accounted for about 75% and SBP for about 20% of the PWV variance, which together accounted for 99.1% of the total PWV variance [23].

### Limitations

Data were collected in a single-center registry analysis of a prospective kept database in routine clinical practice. PWV was determined with only one method, namely the Mobil-O-Graph. No comparison was made with other non-invasive methods, nor was echocardiography available. Patients were predominantly treated endovascularly, so a comparison with the open surgical procedure was not meaningful. The number of patients in some age groups was small. Strengths of the present data analysis include the comparison of the PWV in a collective of 381 patients with aortic disease and nonaortic atherosclerotic vascular disease and, in contrast to other studies, a high proportion of patients 70 years and older (172/381, 45.1%). The data were collected by a single, specially trained person with the fully automatic oscillometric Mobil-O-Graph device.

## 5. Conclusions

The Mobil-O-Graph was suitable for simple and valid measurement of blood pressure and PWV in patients with aortic disease. PWV correlated with age and showed a significant dependence on current SBP. Neither aortic disease versus nonaortic AVD, its treatment, nor other cardiovascular risk factors had a significant effect on PWV. As age cannot be influenced, successful blood pressure control is crucial to avoid high PWV and thus increased cardiovascular risk. Therefore, high blood pressure should be addressed in all patients with aortic disease, especially after endovascular aortic repair.

Few studies are available on the increase in PWV and aortic stiffness after endovascular aneurysm repair. Robust evidence to draw conclusions is not yet available. In further studies, it is essential to consider and correct for the effects of age and blood pressure to avoid misinterpretations in comparative PWV analyses.

## Figures and Tables

**Table 1:** Baseline demographics of patients (*n* = 381).

		AoD (*n* = 176) Mean ± SD Number (%)	Nonaortic AVD (*n* = 205) Mean ± SD Number (%)
Age (years)		67 ± 9	68 ± 10
Gender	Male	144 (81.8)	144 (70.2)
	Female	32 (18.2)	61 (29.8)
Antihypertensives		159 (90.3)	170 (82.9)
BMI (kg/m^2^)		28.6 ± 5.5	27.7 ± 5.2
Diabetes		35 (19.9)	75 (36.6)
Smoking		45 (25.6)	44 (21.5)
eGFR		72 ± 25	70 ± 28
Hemodialysis		7 (4.0)	14 (6.8)
AAA		114 (64.8)	0 (0.0)
T(A)AA		37 (21.0)	0 (0.0)
Aortic dissection		25 (14.2)	0 (0.0)
CAD		70 (39.8)	86 (42.0)
PAD		40 (22.7)	141 (68.8)
Carotid AD		27 (15.3)	84 (41.0)

BMI, body mass index; eGFR: estimated glomerular filtration rate; AAA: abdominal aortic aneurysm; T(A)AA: thoracic (thoracoabdominal) aortic aneurysm; CAD: coronary artery disease; PAD: peripheral artery disease; carotid AD: carotid artery disease.

**Table 2:** PWV of patients with different cardiovascular risk factors and degrees of chronic kidney disease (*n* = 381).

Factor	Mean PWV (m/s)	Mean PWV (m/s)	Mean PWV (m/s)	Mean PWV (m/s)	Mean PWV (m/s)	Mean PWV ± SD (m/s)	*p*	p_age corrected_
Age Category	40–49 y	50–59 y	60–69 y	70–79 y	80–90 y	All Patients		
BMI								
<25 kg/m^2^ (*n* = 111)	7.1 (*n* = 5)	7.4 (*n* = 16)	9.2 (*n* = 32)	11.0 (*n* = 45)	12.6 (*n* = 13)	**10.0 ± 1.8**		
25–29.9 kg/m^2^ (*n* = 151)	6.8 (*n* = 7)	8.1 (*n* = 26)	9.3 (*n* = 53)	11.0 (*n* = 56)	12.6 (*n* = 9)	**9.8 ± 1.6**	>0.05	**>0.05**
≥30 kg/m^2^ (*n* = 119)	6.9 (*n* = 4)	8.2 (*n* = 24)	9.5 (*n* = 42)	10.9 (*n* = 37)	12.9 (*n* = 12)	**9.9 ± 1.6**	>0.05	**>0.05**
Diabetes								
Diabetics (*n* = 110)	6.8 (*n* = 1)	8.2 (*n* = 15)	9.2 (*n* = 42)	11.0 (*n* = 41)	12.9 (*n* = 11)	**10.1 ± 1.6**		
Nondiabetics (*n* = 271)	6.9 (*n* = 15)	8.0 (*n* = 51)	9.5 (*n* = 85)	11.0 (*n* = 97)	12.6 (*n* = 23)	**9.9 ± 1.7**	>0.05	**>0.05**
Smoking								
Smokers (*n* = 89)	7.0 (*n* = 3)	8.2 (*n* = 30)	9.3 (*n* = 34)	10.9 (*n* = 19)	12.2 (*n* = 3)	**9.3 ± 1.4**		
Nonsmokers (*n* = 292)	6.8 (*n* = 13)	8.0 (*n* = 36)	9.4 (*n* = 93)	11.0 (*n* = 119)	12.8 (*n* = 31)	**10.1 ± 1.7**	<0.05	**>0.05**
eGFR								
≥90 (*n* = 107)	7.0 (*n* = 5)	8.2 (*n* = 32)	9.4 (*n* = 48)	11.1 (*n* = 20)	12.1 (*n* = 2)	**9.2 ± 1.3**		
30–89 (*n* = 244)	6.7 (*n* = 9)	8.0 (*n* = 29)	9.3 (*n* = 68)	11.0 (*n* = 110)	12.7 (*n* = 28)	**10.2 ± 1.7**	<0.05	**>0.05**
<30 (*n* = 30)	7.2 (*n* = 2)	8.0 (*n* = 6)	9.5 (*n* = 7)	10.7 (*n* = 11)	13.0 (*n* = 4)	**10.0 ± 1.8**	<0.05	**>0.05**

y, years; BMI, body mass index; eGFR: estimated glomerular filtration rate.

## Data Availability

The original data are pseudonymized and therefore not available online. They are stored at the University Hospital Regensburg.

## References

[1] O’Rourke M.F., Staessen J.A., Vlachopoulos C., Duprez D., Plante G.E. (2002). Clinical applications of arterial stiffness; definitions and reference values. Am. J. Hypertens..

[2] Mihalcea D.J., Florescu M., Suran B.M., Enescu O.A., Mincu R.I., Magda S., Patrascu N., Vinereanu D. (2016). Comparison of pulse wave velocity assessed by three different techniques: Arteriograph, Complior, and Echo-tracking. Heart Vessels.

[3] Townsend R.R., Wilkinson I.B., Schiffrin E.L., Avolio A.P., Chirinos J.A., Cockcroft J.R., Heffernan K.S., Lakatta E.G., McEniery C.M., Mitchell G.F. (2015). Recommendations for Improving and Standardizing Vascular Research on Arterial Stiffness: A Scientific Statement from the American Heart Association. Hypertension.

[4] Reference Values for Arterial Stiffness’ Collaboration (2010). Determinants of pulse wave velocity in healthy people and in the presence of cardiovascular risk factors: ‘establishing normal and reference values’. Eur. Heart J..

[5] Milan A., Zocaro G., Leone D., Tosello F., Buraioli I., Schiavone D., Veglio F. (2019). Current assessment of pulse wave velocity: Comprehensive review of validation studies. J. Hypertens..

[6] Weber T., Wassertheurer S., Rammer M., Maurer E., Hametner B., Mayer C.C., Kropf J., Eber B. (2011). Validation of a brachial cuff-based method for estimating central systolic blood pressure. Hypertension.

[7] Karpetas A., Sarafidis P.A., Georgianos P.I., Protogerou A., Vakianis P., Koutroumpas G., Raptis V., Stamatiadis D.N., Syrganis C., Liakopoulos V. (2015). Ambulatory recording of wave reflections and arterial stiffness during intra- and interdialytic periods in patients treated with dialysis. Clin. J. Am. Soc. Nephrol..

[8] Papaioannou T.G., Thymis J., Benas D., Triantafyllidi H., Kostelli G., Pavlidis G., Kousathana F., Katogiannis K., Vlastos D., Lambadiari V. (2019). Measurement of central augmentation index by three different methods and techniques: Agreement among Arteriograph, Complior, and Mobil-O-Graph devices. J. Clin. Hypertens..

[9] Dogdus M., Akhan O., Ozyasar M., Yilmaz A., Altintas M.S. (2018). Evaluation of Arterial Stiffness Using Pulse Wave Velocity and Augmentation Index in Patients with Chronic Venous Insufficiency. Int. J. Vasc. Med..

[10] Sarafidis P.A., Loutradis C., Karpetas A., Tzanis G., Piperidou A., Koutroumpas G., Raptis V., Syrgkanis C., Liakopoulos V., Efstratiadis G. (2017). Ambulatory Pulse Wave Velocity Is a Stronger Predictor of Cardiovascular Events and All-Cause Mortality Than Office and Ambulatory Blood Pressure in Hemodialysis Patients. Hypertension.

[11] Golebiowski T., Kusztal M., Konieczny A., Letachowicz K., Gawrys A., Skolimowska B., Ostrowska B., Zmonarski S., Janczak D., Krajewska M. (2020). Disability of Dialysis Patients and the Condition of Blood Vessels. J. Clin. Med..

[12] Durmus I., Kazaz Z., Altun G., Cansu A. (2014). Augmentation index and aortic pulse wave velocity in patients with abdominal aortic aneurysms. Int. J. Clin. Exp. Med..

[13] Paraskevas K.I., Bessias N., Psathas C., Akridas K., Dragios T., Nikitas G., Andrikopoulos V., Mikhailidis D.P., Kyriakides Z.S. (2009). Evaluation of aortic stiffness (aortic pulse-wave velocity) before and after elective abdominal aortic aneurysm repair procedures: A pilot study. Open Cardiovasc. Med. J..

[14] Hannuksela M., Johansson B., Carlberg B. (2018). Aortic stiffness in families with inherited non-syndromic thoracic aortic disease. Scand. Cardiovasc. J..

[15] Lee C.W., Sung S.H., Chen C.K., Chen I.M., Cheng H.M., Yu W.C., Shih C.C., Chen C.H. (2013). Measures of carotid-femoral pulse wave velocity and augmentation index are not reliable in patients with abdominal aortic aneurysm. J. Hypertens..

[16] Gray C., Goodman P., Badger S.A., O’Malley M.K., O’Donohoe M.K., McDonnell C.O. (2016). Endovascular Aneurysm Repair Increases Aortic Arterial Stiffness When Compared to Open Repair of Abdominal Aortic Aneurysms. Vasc. Endovascular. Surg..

[17] Tzilalis V.D., Kamvysis D., Panagou P., Kaskarelis I., Lazarides M.K., Perdikides T., Prassopoulos P., Boudoulas H. (2012). Increased pulse wave velocity and arterial hypertension in young patients with thoracic aortic endografts. Ann. Vasc. Surg..

[18] Youssef A., Kalaja I., Alkomi U., Abt T., Hoffmann R.T., Reeps C., Weiss N., Karl Lackner H., Mahlmann A. (2020). Aortic stiffness and related complications after endovascular repair of blunt thoracic aortic injury in young patients. Vasa.

[19] Moloney M.A., McHugh S., DH O.D., Casey R.G., Kavanagh E.G., Grace P.A., Fitzgerald P., Bouchier-Hayes D.J. (2011). Comparison of arterial stiffness and microcirculatory changes following abdominal aortic aneurysm grafting. Ir. J. Med. Sci..

[20] Bissacco D., Conti M., Domanin M., Bianchi D., Scudeller L., Mandigers T.J., Allievi S., Auricchio F., Trimarchi S. (2022). Modifications in Aortic Stiffness after Endovascular or Open Aortic Repair: A Systematic Review and Meta-Analysis. Eur. J. Vasc. Endovasc. Surg..

[21] Holda M.K., Iwaszczuk P., Wszolek K., Chmiel J., Brzychczy A., Trystula M., Misztal M. (2020). Coexistence and management of abdominal aortic aneurysm and coronary artery disease. Cardiol. J..

[22] Hernesniemi J.A., Vanni V., Hakala T. (2015). The prevalence of abdominal aortic aneurysm is consistently high among patients with coronary artery disease. J. Vasc. Surg..

[23] Schwartz J.E., Feig P.U., Izzo J.L. (2019). Pulse Wave Velocities Derived from Cuff Ambulatory Pulse Wave Analysis. Hypertension.

[24] Ben-Shlomo Y., Spears M., Boustred C., May M., Anderson S.G., Benjamin E.J., Boutouyrie P., Cameron J., Chen C.H., Cruickshank J.K. (2014). Aortic pulse wave velocity improves cardiovascular event prediction: An individual participant meta-analysis of prospective observational data from 17,635 subjects. J. Am. Coll. Cardiol..

[25] Bia D., Zocalo Y. (2021). Physiological Age- and Sex-Related Profiles for Local (Aortic) and Regional (Carotid-Femoral, Carotid-Radial) Pulse Wave Velocity and Center-to-Periphery Stiffness Gradient, with and without Blood Pressure Adjustments: Reference Intervals and Agreement between Methods in Healthy Subjects (3–84 Years). J. Cardiovasc. Dev. Dis..

[26] Salvi P., Scalise F., Rovina M., Moretti F., Salvi L., Grillo A., Gao L., Baldi C., Faini A., Furlanis G. (2019). Noninvasive Estimation of Aortic Stiffness Through Different Approaches. Hypertension.

[27] Hametner B., Wassertheurer S., Kropf J., Mayer C., Eber B., Weber T. (2013). Oscillometric estimation of aortic pulse wave velocity: Comparison with intra-aortic catheter measurements. Blood Press. Monit..

[28] Brett S.E., Guilcher A., Clapp B., Chowienczyk P. (2012). Estimating central systolic blood pressure during oscillometric determination of blood pressure: Proof of concept and validation by comparison with intra-aortic pressure recording and arterial tonometry. Blood Press. Monit..

[29] Papaioannou T.G., Argyris A., Protogerou A.D., Vrachatis D., Nasothimiou E.G., Sfikakis P.P., Stergiou G.S., Stefanadis C.I. (2013). Non-invasive 24 hour ambulatory monitoring of aortic wave reflection and arterial stiffness by a novel oscillometric device: The first feasibility and reproducibility study. Int. J. Cardiol..

[30] Sarafidis P.A., Georgianos P.I., Karpetas A., Bikos A., Korelidou L., Tersi M., Divanis D., Tzanis G., Mavromatidis K., Liakopoulos V. (2014). Evaluation of a novel brachial cuff-based oscillometric method for estimating central systolic pressure in hemodialysis patients. Am. J. Nephrol..

[31] Li H., Lin K., Shahmirzadi D. (2016). FSI Simulations of Pulse Wave Propagation in Human Abdominal Aortic Aneurysm: The Effects of Sac Geometry and Stiffness. Biomed. Eng. Comput. Biol..

